# 785. British Transplant ID, Mycology, and Infection in the Immune Compromised Network (BRITMIC) -Addressing the unmet need of Transplant ID in the United Kingdom

**DOI:** 10.1093/ofid/ofad500.846

**Published:** 2023-11-27

**Authors:** Neil R H Stone, Saraswathi Murthy, Emmanuel Wey, Jonathan Lambourne

**Affiliations:** University College London Hospitals, London, England, United Kingdom; Guy's and St Thomas' NHS Foundation Trust, London, England, United Kingdom; Royal Free Hospital, London, London, England, United Kingdom; Barts Health NHS Trust, London, England, United Kingdom

## Abstract

**Background:**

There is currently no formal sub-specialty training program in transplant ID or mycology in the UK. Practice is diverse and is provided by either ID physicians or clinical microbiologists with a self declared interest in these aspects of infection medicine. To address this, we established a new network is designed to promote this ever expanding niche within the wider specialty of infectious diseases and microbiology

**Methods:**

British Transplant ID, Mycology, and Infection in the Immune Compromised Network (BRITMIC) was established in 2021 as a UK-wide network for infection specialists with an interest in clinical mycology, infections in solid and stem cell transplants and infections in the immunocompromised host. Our aim is to share interest and experience in invasive fungal infections and transplant infectious diseases, and provide a platform for communication, education and research, as well as real-time clinical case discussions.

**Results:**

Since establishment we have 117 registered members and have run quarterly meetings (currently virtual), with our inaugural meeting having been held in July 2021. We have held 10 meetings to date had outstanding international guest speakers, complex case discussions, as well as hosting a highly successful session on Transplant ID at the Federation of Infection Societies (FIS) Conference in November 2021. We are now affiliated to the larger British Infection Association (BIA) and have an established governance framework and terms of reference.

Achievements to date include developing a UK working group on treatments for COVID-19 in the immune suppressed and establishing a national case conference for complex mycology or transplant related infections.

**Conclusion:**

The BRITMIC Network has established a new resource for mycology and transplant ID in the UK, and is an important stepping stone to establish these as recognised sub specialties within the field of ID in our setting. This can act as a model for others in settings with limited existing networking and education infrastructure in this branch of ID

**Disclosures:**

**Neil RH Stone, MD PhD**, Gilead: Advisor/Consultant|Gilead: Grant/Research Support|Gilead: Honoraria|Pfizer: Honoraria|Shionogi: Honoraria **Emmanuel Wey, MBBS**, Clover Biosoft: Advisor/Consultant **Jonathan Lambourne, MB BS, BA, MSc, DTM&H FRCP, FRCPath, PhD**, Mundipharma: Advisor/Consultant|Mundipharma: Advisor/Consultant

